# Neutrophil extracellular traps are found at epithelial barriers in canine sinonasal aspergillosis

**DOI:** 10.3389/fimmu.2026.1808191

**Published:** 2026-05-01

**Authors:** Pierre Janssen, Constance de Meeûs, Mutien-Marie Garigliany, Cecile Clercx, Thomas Marichal, Coraline Radermecker, Frederic Billen

**Affiliations:** 1 Laboratory of Immunophysiology, Grappe Interdisciplinaire de Génoprotéomique Appliquée-Recherche (GIGA-R), Liege University, Liege, Belgium; 2 Faculty of Veterinary Medicine, Liege University, Liege, Belgium; 3 Laboratory of Animal Pathology, INstitut DEs EPidémies (INDEEP), Liege University, Liege, Belgium; 4 Department of Clinical Companion Animal Services, Liege University, Liege, Belgium; 5 Walloon Excellence in Life Sciences and Biotechnology (WELBIO) Department, Walloon Excellence in Life Sciences (WEL) Research Institute, Wavre, Belgium

**Keywords:** dogs, nasal mucosa, neutrophil, neutrophil extracellular traps, sino-nasal aspergillosis

## Abstract

**Introduction:**

Sinonasal aspergillosis (SNA) is a frequent cause of chronic nasal discharge, predominantly caused by *Aspergillus fumigatus*. The infection is typically confined to the nasal cavity and/or frontal sinuses, characterized by superficial mucosal fungal plaques and associated with severe lymphoplasmacytic and neutrophilic inflammation of the mucosa. While Neutrophil Extracellular Traps (NETs) have been demonstrated to restrict fungal hyphal proliferation in animal models, their presence and role in canine SNA remain unexplored.

**Methods:**

In this study, we quantified the presence of NETs in nasal lavage fluid (NALf) or nasal mucosal biopsies from dogs diagnosed with SNA and healthy controls. To unambiguously detect NETs, we employed three detection techniques. The first method consisted in cell-free DNA detection in NAL. The second method measured myeloperoxidase (MPO)-DNA complexes in NAL by ELISA. The third method aimed to directly identify NETs in nasal biopsies by confocal microscopy and characterize histopathological lesions associated to NETs-enriched areas in corresponding adjacent hematoxylin-eosin tissue sections.

**Results:**

Interestingly, we were only able to detect NETs in biological samples of dogs with SNA but not in samples from control dogs. Furthermore, NETs-enriched areas were specifically restricted to the surface of the epithelial barrier, where they surrounded fungal spots.

**Conclusion:**

These results suggest that NETs are involved in the inflammatory response occurring in SNA and likely regulate disease progression. Whether they limit the fungal propagation or contribute to the pathogenesis of the disease remains to be determined, before assessing their role as potential therapeutic target.

## Introduction

Phagocytic cells of the innate immune system serve as the first line of defense against invading fungal organisms (1). Among these, neutrophils represent the most abundant innate effector cells involved in fungal infection control, employing multiple antifungal mechanisms, including phagocytosis, degranulation, and the production of reactive oxygen species (2). Neutrophils are easily identifiable innate immune cells characterized by their multilobed nuclei and cytoplasmic granules, which store cytotoxic compounds (3). They are among the first immune cells recruited to sites of infection, where they limit pathogen dissemination through potent phagocytic activity, cytotoxic granule release, and the deployment of Neutrophil Extracellular Traps (NETs) (4), ultimately facilitating extracellular fungal elimination (2). NETs are extracellular structures composed of nuclear or mitochondrial DNA, associated with citrullinated histone 3, and decorated with various antimicrobial peptides, including myeloperoxidase (MPO), neutrophil elastase (NE), LL-37, and cathelicidins (4). NETs can be released *in vivo* in response to diverse stimuli, such as bacteria (5), viruses (6–8), parasites (9, 10), immune complexes (11), and crystals (12). They function to trap pathogens, preventing systemic dissemination, while also exerting direct antimicrobial effects (4). Despite their protective role, excessive NET production or impaired NET clearance have been implicated in immune-mediated disorders, including rheumatoid arthritis (13), ANCA-associated vasculitis (14), atherosclerosis (15), and coagulopathies (16, 17).

In dogs, sino-nasal aspergillosis (SNA) represents one of the most common causes of chronic nasal discharge, predominantly caused by *A. fumigatus* (18). The infection remains localized to the nasal cavity and/or frontal sinus and is characterized by superficial mucosal fungal plaques associated with severe lymphoplasmacytic and neutrophilic inflammation (19). Of note, *A. fumigatus* is an ubiquitous fungus, affected dogs are not systemically immunocompromised and the infection remains confined to the nasal and/or frontal sinuses. Hence, a localized nasal mucosal immune dysfunction is suspected to contribute to the pathogenesis of SNA (18). While an imbalance between type 1 and type 17 immune responses has been proposed to perpetuate infection and the associated inflammatory response (20), additional mechanisms underlying the failure to clear fungal infection remain to be explored. Notably, the role of NETs in canine respiratory diseases has not yet been investigated (21).

Studies using animal models of pulmonary aspergillosis have highlighted an important contribution of NETs to the containment and clearance of fungus (22). Neutrophils have been shown to generate NETs upon encountering *Aspergillus fumigatus*, with a more pronounced NETs release in response to the larger hyphal form compared to smaller conidia (23). The reduced NET release triggered by conidia is associated with the presence of hydrophobin RodA, a major conidial surface component, which likely masks pathogen-associated molecular patterns on the conidial cell wall (24). Furthermore, microbial size is a critical determinant in the sensing mechanism that enables neutrophils to selectively modulate their antimicrobial responses according to pathogen dimensions. Phagocytosis is a rapid process, allowing neutrophils to internalize the maximal number of yeast particles within 30–40 minutes, whereas NETosis is markedly slower, requiring approximately four hours after contact with hyphal structures (25). While fungal hyphae of *A. fumigatus* induce NETs formation, NETs appear to primarily restrict fungal spread rather than directly eliminate the hyphae (24, 26). Finally, biofilm formation by *A. fumigatus* has been suggested as a strategy to evade the antifungal effects of NETs (27).

While the role of NETs in various diseases has been well established in human medicine and mouse models (28), their presence and function in canine species remain unexplored. Additionally, the possibility that NETs could represent a therapeutic target for SNA has not yet been investigated.

In this study, we assessed NETs release in dogs with SNA and examined whether NETs levels correlated with disease severity and treatment response. We first optimized three NET detection methods, which were previously validated in horses: quantification of cell-free DNA and MPO–DNA complexes in nasal lavage fluids, and direct visualization of NETs in nasal biopsies by confocal microscopy combined with histopathological evaluation (29). Using these methods, we compared NET presence in nasal lavage and mucosal biopsies from healthy dogs, dogs with non-fungal chronic nasal disease, and dogs with SNA at the time of diagnosis and after treatment.

## Materials and methods

### Animals

Owners gave informed consent for their animals’ inclusion in the study. All animals included in this study were seen at the Small Animal Clinic of the Veterinary Medicine of the University of Liège, between May 2022 and May 2024.

Thirty-nine dogs were included in the study: 28 dogs with SNA and 11 control dogs. Among the diseased dogs, 24 were newly diagnosed with SNA (initial SNA). Six dogs with persistent SNA at the first follow-up visit were also enrolled; three of these had already been included in the initial SNA group. In addition, two dogs with resolved SNA were included, one of which had previously been part of the initial SNA group (**Table 1**).

**Table 1:** Clinical characteristics and diagnosis findings of SNA aspergillosis and control dogs in this study.

Group	Breed	Age	Sex	Wt	Dur	Disch	Epis	Syst	Side	CT	Rhino	Cult	PCR
Initial	Mixed	3	NM	23	3	MP	✓	–	R	✓	FP	✓	–
Initial	Border Collie	8	M	30	3	MP	✓	✓	L	✓	FP	✓	–
Initial	Golden retriever	9	SF	38	2	MP	✓	✓	R	✓	FP	✓	–
Initial	American Cocker Spaniel	10	M	13	3	MP	–	✓	L	✓	FP	✓	–
Initial	German Shepherd	3	M	33	7	–	✓	✓	R	✓	FP	✓	–
Initial	Golden retriever	10	M	24	1	MP	✓	✓	R+L	✓	FP	✓	–
Initial	Bull Terrier	4	SF	20	2	–	✓	–	L	✓	FP	–	✓
Initial	Mixed	10	M	21	3	MP	✓	✓	R	✓	FP	✓	–
Initial	Australian Shepherd	1	M	22	3	–	✓	✓	L	✓	FP	✓	–
Initial	Belgian Shepherd	12	M	20	7	MP	✓	✓	R	✓	FP	✓	✓
Initial	American staffordshire Terrier	10	NM	24	12	MP	–	–	L+R	✓	FP	✓	–
Initial	Jagd Terrier	2	NM	8	2	MP	✓	–	R	✓	FP	✓	–
Initial	Hovawart	4	M	37	2	MP	–	✓	L	–	FP	✓	–
Initial	Jack Russel Terrier	1	M	8	2	MP	✓	–	R	✓	FP	✓	–
Initial	Husky	8	SF	31	2	MP	–	✓	R+L	✓	FP	✓	–
Initial	Basset Fauve de Bretagne	9	NM	16	8	MP	–	–	R+L	✓	FP	✓	–
Initial	Labrador Retriever	11	M	32	5	MP	–	✓	L+R	✓	FP	✓	–
Initial	Golden Retriever	5	SF	44	4	–	✓	✓	L	–	FP	✓	–
Initial	Rottweiler	7	SF	44	1	MP	✓	✓	L	–	FP	–	✓
Initial	Border Collie	7	M	31	2	MP	✓	✓	R	–	FP	✓	–
Initial	Mixed	6	NM	29	6	MP	✓	✓	R	✓	FP	✓	–
Initial	Rottweiler	7	SF	42	2	MP	✓	✓	L	✓	FP	✓	–
Initial	Labrador Retriever	10	SF	33	3	MP	–	–	R	✓	FP	–	✓
Initial	Belgian Shepherd	12	SF	21	3	MP	✓	–	R	–	FP	✓	–
Persistent	Mixed	3	NM	23	3	MP	✓	–	R	–	FP	✓	–
Persistent	German Shepherd	3	M	33	7	MP	–	–	R	–	FP	✓	–
Persistent	Bull Terrier	4	M	20	2	S	–	–	R+L	–	FP	–	✓
Persistent	Hovawart	4	M	40	4	–	–	–	L	–	FP	✓	–
Persistent	Great dane	6	ME	82	2	MP	–	–	L	–	FP	✓	–
Persistent	Husky	8	SF	31	2	–	–	–	R+L	–	FP	✓	–
Resolved	Golden Retriever	7	M	34	1	S	–	–	R+L	–	clean	nan	–
Resolved	Jack Russel Terrier	1	M	8	2	–	–	–	R	–	clean	nan	–

NM, Neutered Male; M, Male; SF, Spayed Female; Wt, Body weight (kg); Dur, Duration (months); Disch, Nasal discharge (MP, mucopurulent; S, serous); Epis, Epistaxis; Syst, Systemic signs; Side, L, left; R, right; R>L, bilateral; Rhino, Rhinoscopy (FP, fungal plaques); Cult, Fungal culture; PCR, Aspergillus PCR; Yes/Positive; -: No/Negative/Not available.

SNA was diagnosed based on history, clinical signs, diagnostic imaging and the observation of typical endoscopic findings such turbinate destruction associated with the presence of fungal plaques in the frontal sinus and/or nasal cavity. Eleven control dogs euthanized for extra-nasal non-infectious diseases were also included in the study. The causes for euthanasia were pulmonary hemorrhage (n=1), idiopathic pulmonary fibrosis (n=1), intervertebral disk hernia (n=1), AKI (n=1) and cancers of mammary glands (n=2), liver (n=1), abdomen (n=1), brain (n=1), heart (n=1) and bone (n=1).

### Reagents and antibodies

A complete list of the reagents, antibodies and software used in this manuscript can be found in **Table 2**.

**Table 2:** List of antibodies, reagents and software used in this study.

Antibodies	Source	Cat#
3,3’,5,5’ Tétraméthylbenzidine (TMB)	Life technologies	SB02
4’,6-diamidino-2-phénylindole (DAPI)	ThermoFisher	D3571
Ammonium chloride (NH_4_Cl)	VWR chemicals	21236267
Anti-goat IgG (H+L) Polyclonal Antibody (Donkey), AF488 conjugated	ThermoFisher	11055
Anti-mouse DNA Monoclonal Antibody (Rat, clone BV16-13), unconjugated	Sigma Aldrich	MAB030
Anti-mouse IgG2a Biotin Antibody (Rat)	BD Biosciences	553388
Anti-mouse/human Histone H3 (citrulline R2+R8+R17) Polyclonal Antibody (Rabbit), Unconjugated	Abcam	Ab5103
Anti-mouse/human Myeloperoxidase/MPO Polyclonal Antibody (Goat), Unconjugated	R&D Systems	AF3637
Anti-rabbit IgG (H+L) Polyclonal Antibody (Donkey), AF568 conjugated	ThermoFisher	A10042
Avidin-HRP	ThermoFisher	18-4100-94
Bovine Serum Albumin (BSA)	Sigma	A7906
Chemicals, Peptides and Recombinant Proteins	Source	Cat#
Detomidine (Domidine^®^)	Alcyon	2401677
DMEM, low glucose, pyruvate, no glutamine, no phenol red	Gibco	11580406
DNase I	Sigma	11284932001
dNTP	ThermoFisher	N8080260
DPBS	ThermoFisher	14190094
EDTA	Merck Millipore	1084181000
Fetal Bovine Serum (FBS)	Sigma	F7524
Formaldehyde 37%	Fisher	10532955
Hematoxylin and Eosin Stain Kit	Bio-Techne	H-3502-NB
Periodic Acid-Schiff (PAS) Staining System	Sigma-Aldrich	395B
ProLong™ Diamond Antifade Mountant	ThermoFisher	P36961
Quant-iT™ PicoGreen™ dsDNA Assay Kit	ThermoFisher	P11496
Silver Stain (Modified GMS) Kit	Sigma-Aldrich	HT100A
Triton X-100 Detergent	Merck	648466
Tween-20	ThermoFisher	233360010
Software and algorithms	Source	URL
Adobe Illustrator 2022	Adobe	
Prism 9	GraphPad Software	https://www.graphpad.com/scientific-software/prism/
QuPath	QuPath Software	https://qupath.github.io

### Sample collection and processing

Samples of nasal lavage fluid (NALf) and mucosal nasal biopsies were collected from all dogs.

NAL was performed as described earlier by Biénès (30). The diseased dogs were anesthetized using butorphanol (0.2 mg/kg; Butomidor^®^, Richter Pharma, Wels, Austria) in combination with medetomidine (5 μg/kg; Medetor^®^, CP-Pharma, Burgdorf, Germany) intravenously. Propofol (2–4 mg/kg to effect; Propovet^®^, Zoetis, Malakoff, France) intravenously was used for induction. Anesthesia was maintained with isoflurane (Iso-Vet^®^; Eurovet, Bladel, Netherlands), for endoscopic evaluation of the respiratory tract, intubated and placed in ventral recumbency. The dogs that were euthanized and used as controls received initially a similar protocol with butorphanol (0.2 mg/kg; Butomidor^®^, Richter Pharma, Silures, Austria) in combination with medetomidine (5 μg/kg; Medetor^®^, CP-Pharma, Burgdorf, Germany) intravenously, followed by induction with propofol (2–4 mg/kg to effect; Propovet^®^, Zoetis, Malakoff, France). Finally, euthanasia was performed with intravenous injection of pentobarbital (100–150 mg/kg; Euthasol^®^Vet, Dechra, Bladel, Netherlands). In all dogs, the nasopharynx was manually blocked. A multi-fenestrated catheter connected to a 60 ml syringe was introduced into the first third of the most clinically affected nasal cavity. NAL was performed by vigorously injection of +/- 1 ml/kg of sterile isotonic saline solution via the nasal catheter, while both nostrils being manually occluded to limit leakage of the saline. The fluid was directly retrieved via manual aspiration with the same syringe.

A differential cell count was performed on 3 x 10^2^ cells; each cell type was counted as a percentage of total nucleated cells. The remaining NALf was centrifuged twice for 15 minutes in a countertop centrifuge at 2,500 rpm (541 x g, rotor S-4–104 Centrifuge 5810R, Eppendorf, at 4 °C brake 9, accel 9). Supernatants were stored in 1.5 mL aliquots at -80 °C. All samples were processed within 2 hours of collection.

After performing the NAL, biopsies of affected mucosal areas in SNA dogs or from normal ethmoidal turbinates in control dogs, were taken from the same nasal cavity with biopsy forceps under endoscopic guidance using a rigid endoscope (cystoscope K Storz SL 30°, Karl-Storz-Endoscopy Belgium SA) or a flexible bronchoscope (Fujinon BRO-YP2, Onys SA). All biopsy samples were transported of the Department of Pathology, University of Liege, at room temperature in a sterile container without any additives.

### Blood collection

Venous blood samples were obtained after the NALF procedure by jugular venipuncture using 5-mL plastic syringes and 21-G needles and placed immediately into 5-mL dry tubes. The tubes were centrifuged (1400 rpm, 7 min) to remove the plasma. Serum was collected into 1.5 mL aliquots and stored at -80 °C.

### Quantification of cf DNA

To estimate the abundance of cf DNA in the supernatant, we used the Quant-iT PicoGreen dsDNA Assay Kit (Invitrogen, Carlsbad, CA, P7589) according to the manufacturer’s instructions. Briefly, a DNA standard curve (from 0.4 µg/mL to 3.125.10^-3^ µg/mL) was performed to estimate double-stranded (ds)DNA concentration of the samples. Samples were diluted 20x and Quant-IT PicoGreen reagent was added to the wells. Fluorescence signals were detected on the Infinite 200 PRO multimode plate reader (Tecan Group Ltd., Switzerland) with filter settings of 485 nm and 535 nm.

### Enzyme-linked immunosorbent assay detecting MPO-DNA complexes

NETs-associated MPO-DNA complexes were quantified in the NALf supernatant and serum using an adapted sandwich ELISA. 96-well flat-bottom plates were coated with anti-human MPO antibody (3,125.10^3^ mg/mL, goat anti-human/mouse MPO, R&D Systems, AF3667) in PBS overnight at 4 °C. The day after, plates were blocked with PBS-Bovine Serum Albumin 1% (BSA, Sigma, A7906), samples (i.e., NALF supernatant or serum) were added together with 1 µL DNase I (RNase free, 125 UI, Sigma, 11284932001). After 15 minutes, we stopped the enzymatic reaction by adding 1 µL PBS-EDTA (0.05 M) and plates were incubated for 90 minutes at room temperature. Mouse anti-DNA detection antibodies (1.10^-2^ µg/mL, clone BV16-13, Sigma-Aldrich, MAB030) were added and incubated for 1 hour. Biotinylated polyclonal rat anti-mouse IgG2a (1.10^-2^ µg/mL, BD Biosciences, 553388) were then added for 90 minutes. Plates were then washed and strepavidin-conjugated horseradish peroxidase (HRP) (1:500 dilution, ThermoFisher, 18-4100-94) was added for 30 minutes. After washing, the plate was incubated in the dark with TMB (3,3’,5,5’ Tétraméthylbenzidine, Lifetechnologies, SB02) substrate, and the enzymatic reaction was stopped with H_2_SO_4–_1 M. Absorbance was measured at 450 nm with a plate reader Multiskan FC (ThermoScientific, 51119000). Between each step until the addition of TMB, 3 to 5 rinses were performed with a wash solution of PBS-Tween-20 5% (ThermoFisher, 233360010).

### Biopsy processing

Following collection, samples were fixed overnight in 4% PFA at 4 °C. After fixation, the tissues were transferred to 75% ethanol and subsequently processed for embedding in paraffin. Serial tissue sections of 4 μm were cut from these blocks for histological and immunofluorescence examination, ensuring uniform processing across all experimental groups.

### Immunofluorescence staining and analysis

To identify NETs from nasal biopsies, after deparaffinization and rehydration, FFPE tissue sections were boiled for 20 min in 10-mM sodium carbonate buffer for antigen retrieval and permeabilized in PBS 0.5% Triton X-100. Samples were then incubated with a blocking buffer (PBS-BSA 2% and FBS 2%; Sigma-Aldrich) for 1 hour at room temperature and stained with rabbit anti-human Cit-H3 (Abcam, Ab5103; 1:100 dilution in blocking buffer) and with goat anti-human MPO (R&D Systems, AF3667; 1:40 dilution in blocking buffer) for 1 hour at room temperature. After washing samples with PBS, secondary donkey anti-rabbit Alexa Fluor 568 (ThermoFisher, A10042, 1:200 dilution in blocking buffer) and donkey anti-goat Alexa Fluor 488 (ThermoFisher, 11055, 1:200 dilution in blocking buffer) were added in blocking buffer containing 4’,6-diamidino-2-phenylindole (DAPI, ThermoFisher, D3571, 1:1000 dilution) and incubated for 2 hours in the dark at room temperature. Finally, samples were mounted with 10 µL of ProLong Antifade reagent (Thermo Fisher, P36961) on glass slides and stored at room temperature in the dark overnight. All samples were analyzed by fluorescence microscopy using standard filter sets. Controls were stained with secondary antibodies after incubation with sera from host species (i.e., rabbit and goat sera, 2% in PBS) without primary antibodies, and nonspecific fluorescent staining was detected under these conditions. Images were acquired on a slice scanner Axioscan 7 from Zeiss and analyzed with QuPath software (version 0.4.3), an open-source software for automated digital pathology analysis.

For NETs identification, we performed a colocalization analysis of triple positive staining for Cit-H3 (red), MPO (green), and DAPI (blue). The colocalization analysis method uses Groovy script. The first step fix 2000 threshold the red Cit-H3, the green MPO staining and the blue DAPI staining. Then the script creates an annotation of the intersection between the Cit-H3, MPO and DAPI staining. Then we created an annotation of the tissue. Finally, we exported the result as a.csv (Comma-separated values) file and we normalized the colocalization area by total area. Background fluorescence was estimated in signal-negative regions for the 488 and 568 channels by measuring the mean fluorescence intensity (MFI) and standard deviation (SD) and calculating a theoretical threshold (MFI + 2×SD, ≈1700); to ensure conservative and consistent analysis, a fixed threshold of 2000 was then manually applied to all images.

The script used to perform confocal microscopy analysis can be found in the following links: https://github.com/AlexHego/Net_Quantification_QuPath.

### Histopathological analysis

Staining with hematoxylin and eosin (HE), periodic acid-Schiff (PAS), and Gomori methenamine silver (GMS) was performed on serial slides containing nasal biopsies. Whole slide images were analyzed using QuPath (version 0.4.3). Lesional areas containing fungi were manually identified on hematoxylin and eosin (HE) and periodic acid-Schiff (PAS) stained slides. Within these regions of interest, superpixels were generated to facilitate the detection of fungal structures. QuPath’s built-in pixel classification algorithms, combined with machine learning, were then used to classify necrotic tissue, fungal spores, and hyphae.

To assess the fluorescence intensity of NETs in these regions, image alignment of PAS-stained and immunofluorescence-stained slides was performed using the Warpy extension in QuPath. This procedure enabled precise spatial alignment between conventional histology and immunofluorescence signals, facilitating direct comparison of tissue morphology and molecular marker distribution within the same anatomical regions. Within the defined regions of interest (ROIs), the mean fluorescence intensity of intersection between the Cit-H3, MPO and DAPI staining was measured on the immunofluorescence-stained slides. The results were reported as the fluorescence intensity of NETs within necrotic tissue compared to regions containing fungal spores and hyphae.

### Statistical analyses

**Figure 1:**
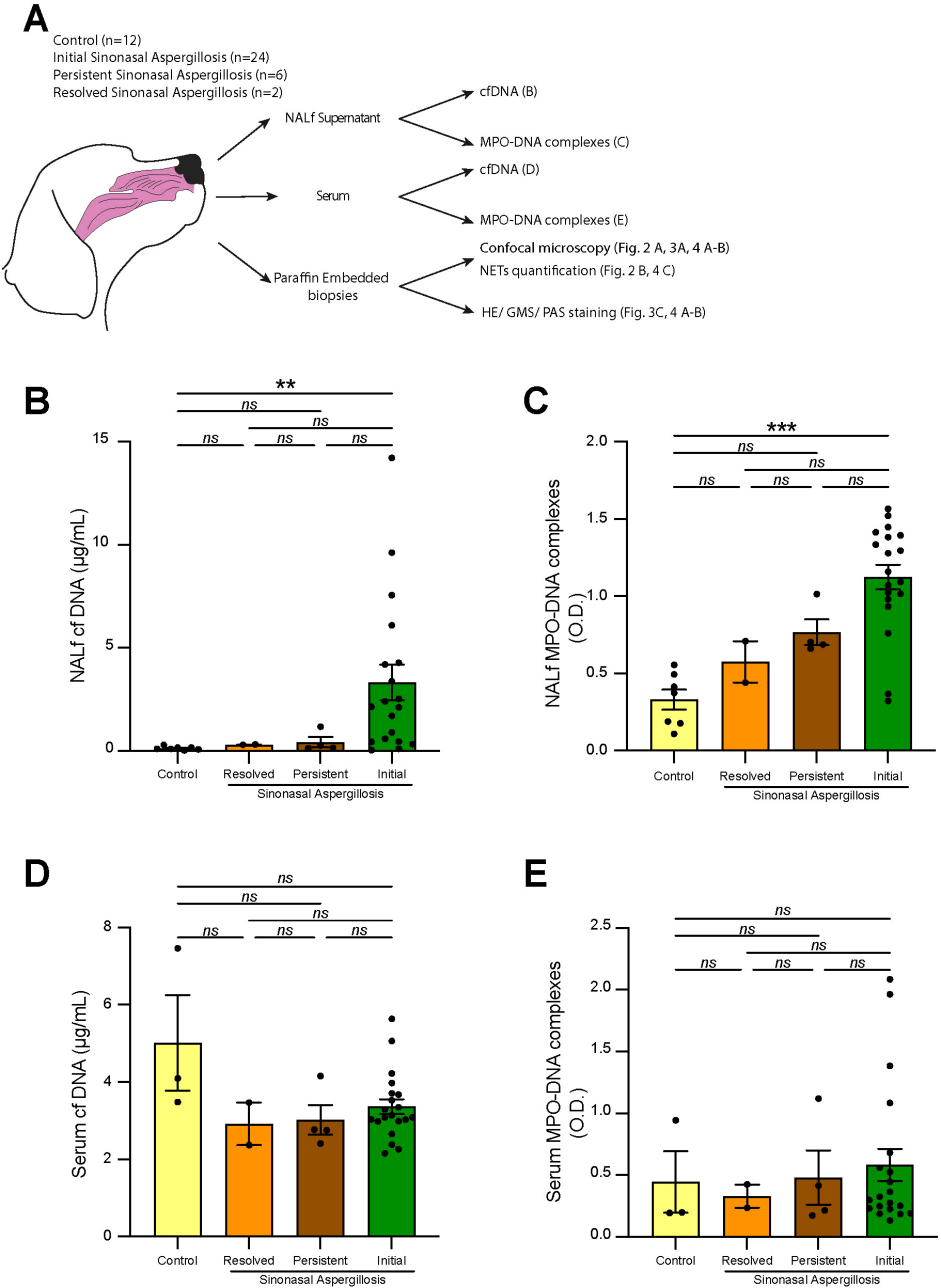
NALf levels of cf DNA, cell free DNA and MPO-DNA complexes in sino-nasal aspergillosis dogs. **(A)** Experimental Outline. NALf was performed, blood serum was collected, biopsy was processed and analyzed by confocal microscopy, while supernatants and serum were recovered to measure cf DNA and MPO-DNA complexes by ELISA. **(B)** NALf cf DNA content measured by PicoGreen assay. **(C)** NALf MPO-DNA complexes measured by ELISA. **(D)** Serum cf DNA content measured by PicoGreen assay. **(E)** Serum MPO-DNA complexes measured by ELISA. **(B–E)** Data show mean + s.e.m., as well as individual values. N NALf Supernatant (control n=7; resolved SNA n=2; persistent SNA n=4, initial SNA n=19). N blood serum (control n=3; resolved SNA n=2; persistent SNA n=4, initial SNA n=20). *P* values were estimated with a one-way ANOVA with Kruskal-Wallis’ *post hoc* test. ^*^P < 0.05; ^**^P < 0.01; ^***^P < 0.001; ns, not significant.

**Figure 2:**
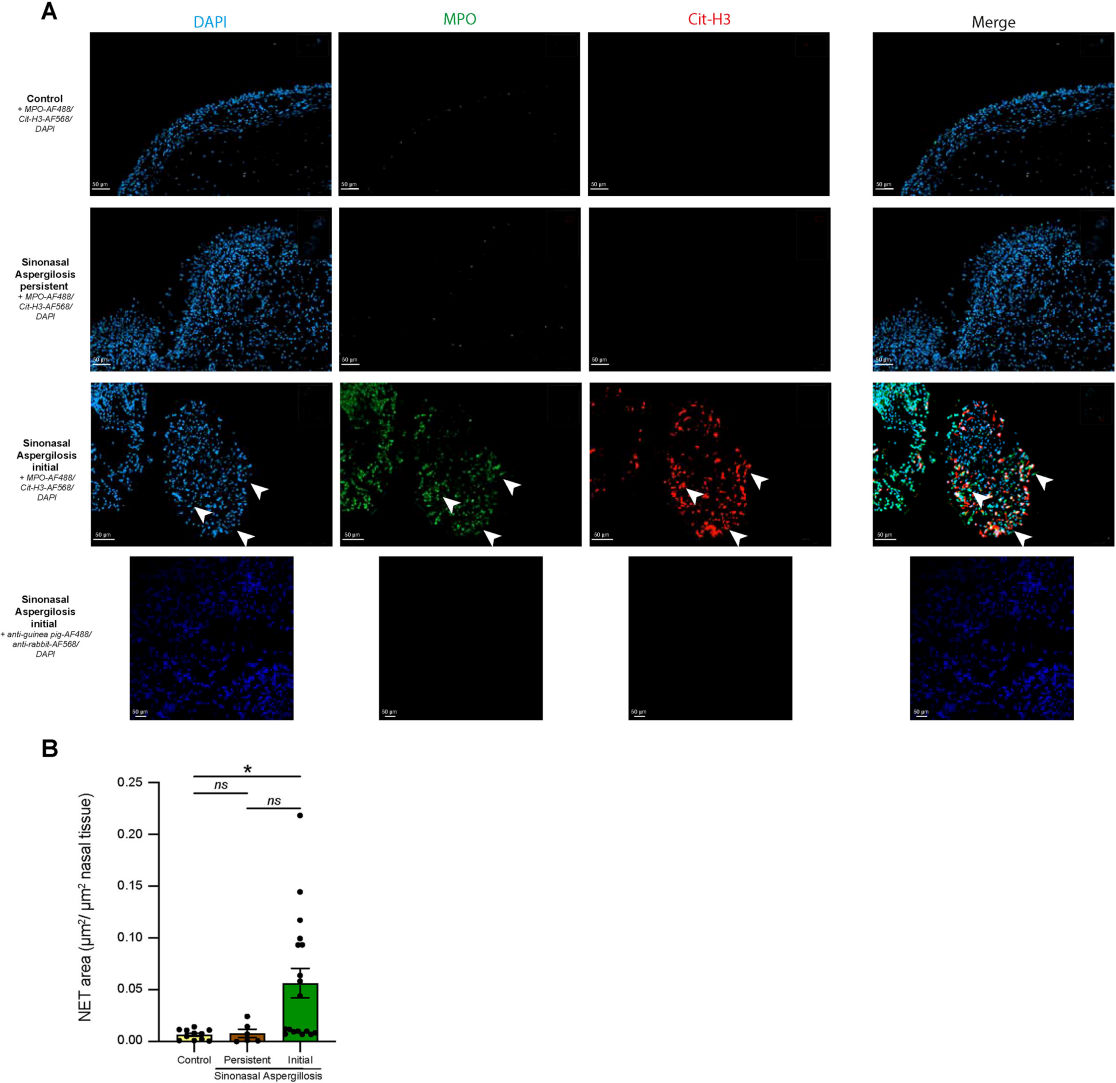
Visualization of NETs release in sino-nasal aspergillosis in dogs. **(A)** Representative confocal microscopy pictures of tissue biopsies stained with anti-MPO and anti-cit-H3 antibodies, and DAPI. NETs are extracellular structures stained positively for MPO and cit-H3 (white arrows). **(B)** NETs quantification in tissue biopsies expressed as NETs area per total tissue area. Data show mean + s.e.m., as well as individual values. (control n=11; persistent SNA n=6, initial SNA n=18). *P* values were estimated with a one-way ANOVA with Kruskal-Wallis’ *post hoc* test. ^*^P < 0.05; ^**^P < 0.01; ^***^P < 0.001; ns, not significant.

The results were analyzed with GrapPad Prism^®^ 10.4.1 (GraphPad Software Inc.). Data in **Figures 1**, **2** are presented as mean + s.e.m. and individual values and were analyzed for statistical significance using a parametric Tukey test or non-parametric Kruskal-Wallis’ test on mean values, as indicated in the respective figure legends. *P* values <0.05 were considered statistically significant. Image processing was done with QuPath software (version 0.5.1).

## Results

### Clinical presentation and classification of dogs

Fungal rhinosinusitis was diagnosed based on compatible clinical signs and evidence of destructive rhinitis on rhinoscopy or CT imaging of the head. Confirmation of a fungal etiology was based on per-endoscopic fungal plaque visualization and fungal culture or quantitative polymerase chain reaction (qPCR) of a fungal plaque or biopsy. All dogs were treated with endoscopic or surgical debridement of the fungal plaques followed by a 15-min enilconazole 2% infusion protocol (31, 32). Control rhinoscopy was performed 3 to 6 weeks after treatment. Cure was based on resolution of clinical signs and absence of fungal plaques. If fungal plaques were still observed, treatment was repeated, and an additional control endoscopy was scheduled 3 to 6 weeks later.

A total of 24 dogs with an initial diagnosis of SNA were retrospectively recruited for this study (16 males, 8 females; aged 7 ± 3.4 years [mean ± SD]). Additionally, 6 dogs with persistent fungal rhinosinusitis at the control visit (5 males, 1 female; aged 4.7 ± 2 years [mean ± SD]) and 2 dogs with resolution of the fungal rhinosinusitis were also included (2 males; aged 5.3 ± 3.8 years [mean ± SD]). Finally, 11 dogs exempt of nasal disease based on history and physical examination, that underwent euthanasia for independent reasons, were prospectively recruited to serve as control dogs (4 males, 7 females; aged 8.8 ± 2.9 years [mean ± SD]) (**Table 3**).

**Table 3:** Overview of distinct biological samples collected according to dog diagnostic categories.

Group	ID	NALf	Serum	Biopsy	cfDNA	MPO-DNA	IF
Control	C1			V			V
Control	C2	V			V	V	
Control	C3	V	V	V	V	V	V
Control	C4	V		V	V	V	V
Control	C5			V			V
Control	C6	V		V	V	V	V
Control	C7	V		V	V	V	V
Control	C8	V	V	V	V	V	V
Control	C9			V			V
Control	C10			V			V
Control	C11	V	V	V	V	V	V
SNA Initial^a^	I1	V	V	V	V	V	V
SNA Initial	I2	V	V	V	V	V	V
SNA Initial	I3	V	V	V	V	V	V
SNA Initial	I4	V	V	V	V	V	V
SNA Initial^b^	I5			V			V
SNA Initial	I6	V	V	V	V	V	V
SNA Initial	I7			V			V
SNA Initial	I8	V	V	V	V	V	V
SNA Initial	I9	V	V	V	V	V	V
SNA Initial	I10	V	V	V	V	V	V
SNA Initial	I11		V	V		V	V
SNA Initial	I12	V	V	V	V	V	V
SNA Initial	I13	V	V	V	V	V	V
SNA Initial^c^	I14	V	V	V	V	V	V
SNA Initial^d^	I15	V	V	V	V	V	V
SNA Initial	I16	V	V	V	V	V	V
SNA Initial	I17	V	V	V	V	V	V
SNA Initial	I18	V	V	V	V	V	V
SNA Initial	I19		V	V			
SNA Initial	I20	V	V		V	V	
SNA Initial	I21	V	V		V	V	
SNA Initial	I22	V			V	V	
SNA Initial	I23	V	V		V	V	
SNA Initial	I24	V		V	V	V	V
SNA Persistent^a^	P1	V	V	V	V	V	V
SNA Persistent^b^	P2	V	V	V	V	V	V
SNA Persistent	P3	V	V	V	V	V	V
SNA Persistent	P4	V	V	V	V	V	V
SNA Persistent	P5			V			V
SNA Persistent^d^	P6			V			V
SNA Resolved	R1	V	V		V	V	
SNA Resolved^c^	R2	V	V	V	V	V	V

Certain subjects were sampled at multiple clinical time points (Initial, Persistent, or Resolved stages), reflecting longitudinal follow-up of the same individuals (marked with identical superscript letters) I1=P1; I5=P2; I14=R2; I15=P6.

SNA, Sinonasal aspergillosis; NALf, Nasal Alveolar Lavage fluid; cfDNA, cell-free DNA; IF, Immunofluorescence.

Analysis of the clinical signs revealed distinct profiles corresponding to the disease status. In the initial SNA group (n=24), clinical signs were typically severe and highly prevalent. The most common finding was a copious mucopurulent (MP) nasal discharge. Epistaxis (nasal bleeding) was also frequently observed (22/24 cases), suggesting significant mucosal and turbinate involvement. Furthermore, systemic signs were reported in most of these dogs (19/24 cases). The localization of the disease was primarily unilateral (14/32 cases at right or 9/32 cases at left), though 9/32 cases with bilateral presentation were also noted. The persistent SNA group (n=6) showed a notable divergence in clinical presentation, often lacking the acute severity seen initially. While nasal discharge remained present and was variable (ranging from mucopurulent to serous), epistaxis and systemic signs were no longer observed in any of the six persistent cases. The localized infection continued to manifest as either unilateral or bilateral nasal involvement. Conversely, dogs categorized as resolved SNA (n=2) exhibited a complete clearance of active clinical pathology. These animals presented no nasal discharge, no epistaxis, and no systemic signs at the time of evaluation. As expected, the control group (n=11), displayed no abnormal clinical signs related to nasal disease (**Table 1**).

### NALf from dogs at initial diagnosis of sinonasal aspergillosis contain elevated levels of cell-free DNA and MPO-DNA complexes

To investigate the presence of NETs in canine SNA, we measured NETs release in NALf from control dogs, dogs with initial SNA, dogs with persistent SNA and dogs with resolved SNA. Cf DNA and MPO-DNA complexes were measured in NALf supernatants. NET quantification by confocal microscopy and *Aspergillus* staining (33) were performed on nasal biopsies (**Figure 1A**). We found significantly higher cf DNA concentrations in NALf from dogs with initial SNA as compared to controls, whereas no differences were observed between controls, resolved SNA (p value >0.9999) and persistent SNA (p value= 0.2977) (**Figure 1B**). Next, we measured MPO-DNA complexes in NALf using an ELISA-based approach. MPO-DNA complexes levels were significantly elevated in the initial SNA group as compared to controls (**Figure 1C**). We observed an increased tendency of MPO-DNA complexes in initial SNA group as compared to resolved SNA (p value= 0.7309) and persistent SNA (p value= 0.6492) even if this did not reach significance (**Figure 1C**). Of note, we did not detect significant differences in cf DNA and MPO-DNA complexes between the control, resolved, and persistent SNA groups. Altogether, our data indicate that cf DNA and MPO-DNA complexes can only be detected at higher levels in the NALf of dogs with initial SNA as compared to healthy dogs. No differences were observed between controls and the persistent and resolved SNA patients, suggesting increased NET formation only during the initial stages of the disease.

We next assessed the presence of NETs in the serum to determine if NET release was strictly restricted to the nasal cavity or if it also involved a systemic component in SNA. Serum samples were processed similarly to NALf samples to evaluate potential systemic NETs release. No differences in serum cf DNA concentration were observed among the different groups (**Figure 1D**). Similarly, ELISA measurements of MPO-DNA complexes revealed no differences between the serum of control dogs, initial SNA, resolved SNA and persistent SNA (**Figure 1E**). Overall, these results suggest that cf DNA and MPO-DNA complexes are not significantly elevated in the systemic circulation of dogs with SNA, indicating that NET formation seems to be restricted to the nasal mucosa without indication of systemic activation of neutrophils associated to NET release.

### NETs are released in nasal tissues of SNA dogs

To further investigate NETs release in tissue samples from dogs with SNA, we performed immunofluorescence stainings to visualize extracellular structures triple positive for DNA (DAPI), citrullinated histone 3 and MPO. Nasal tissue sections from control dogs, dogs with initial or persistent SNA were fixed, permeabilized, and stained with anti-MPO and anti-citrullinated histone H3 (Cit-H3) antibodies, along with DAPI for DNA labelling. Negative controls consisted of tissue sections incubated with secondary antibodies only (**Figure 2A**). We were not able to detect NETs structures in tissue sections from controls and dogs with persistent SNA. In contrast, in biopsies of dogs with initial SNA, we observed numerous extracellular MPO^+^cit-H3^+^DAPI^+^ structures, suggesting active NET release (**Figure 2A**). To quantify NET formation, we measured the total NETs (triple positive signal) area per tissue field and normalized it to the total tissue area (**Figure 2B**). Dogs suffering from initial SNA exhibited a significantly higher NET area as compared to healthy controls and a tendency to increase as compared to dogs suffering from persistent SNA (p value= 0.0624), confirming an increased tendency for NET release in this particular group.

**Figure 3:**
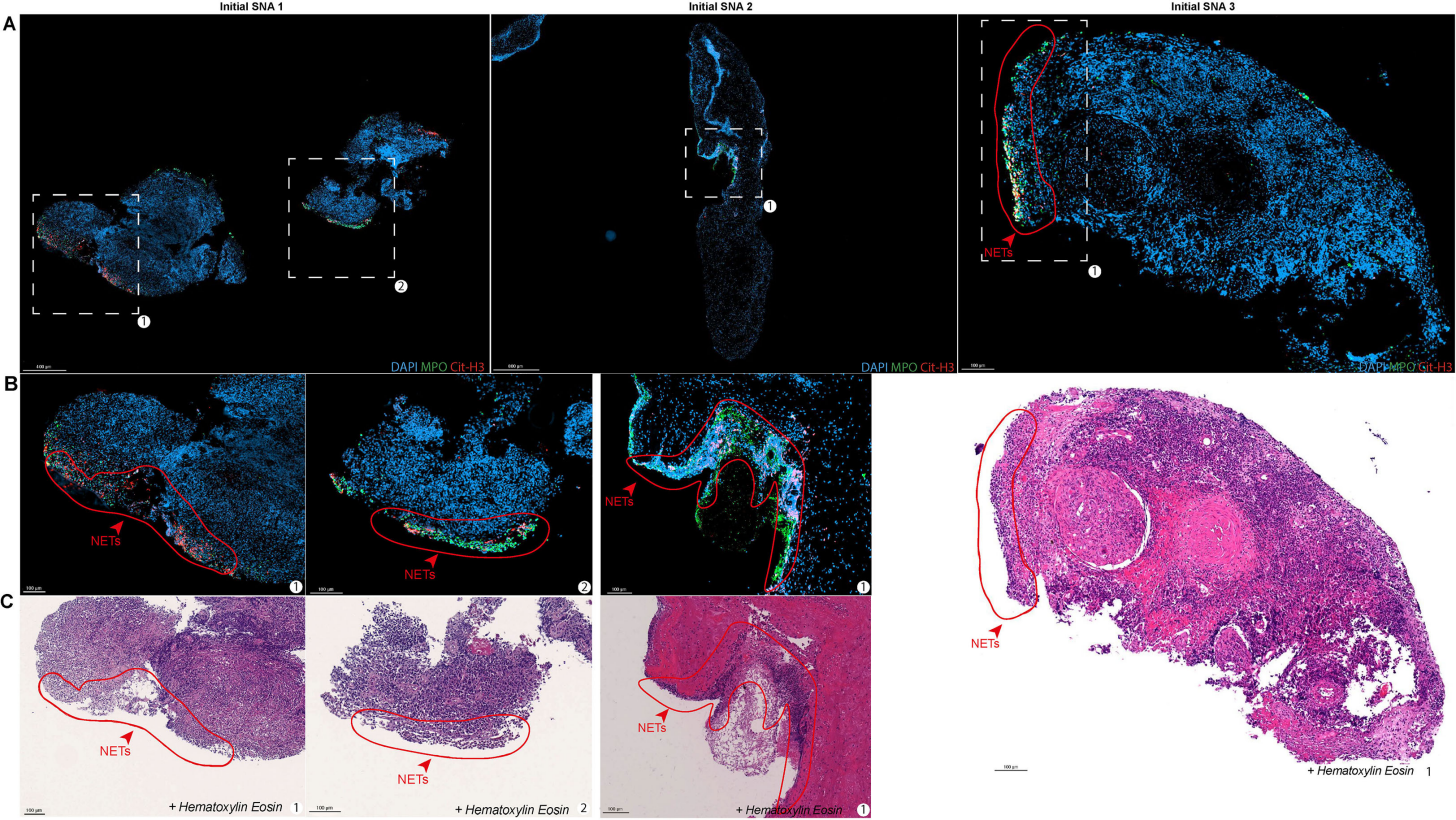
NETs are exclusively detected at biopsy borders. **(A)** Representative immunofluorescence images of whole nasal biopsies (dogs 1-3) showing DNA (DAPI, blue), MPO (green), and Cit-H3 (red). White dashed boxes indicate peripheral regions of interest (ROI). **(B)** High-magnification views of ROIs revealing NETs-enriched areas (red outlines) identified by DNA/MPO/Cit-H3 co-localization at the biopsy edges. **(C)** Corresponding H&E-stained sections highlighting that NET-rich zones. Scale bars appear below each panel.

### NETs are only detected at the epithelial border of nasal biopsies

We next investigated whether NETs present in nasal biopsies of dogs with initial SNA were enriched in specific areas of the nasal mucosa. To this end, serial sections stained with MPO, Cit-H3 and DAPI were analyzed to identify NETs-enriched areas, which were then reported on the corresponding adjacent Hematoxylin-Eosin (H&E)-stained section in order to identify histopathological features associated to NETs. First, immunofluorescence staining revealed the presence of NET-enriched areas located specifically at the periphery of the nasal biopsies (**Figure 3A**). High-magnification imaging of these regions further confirmed the dense accumulation of NET structures (**Figure 3B**). Corresponding H&E staining demonstrated that these NET-rich zones were closely associated with substantial inflammatory (chiefly neutrophilic) cell infiltration, localized hemorrhage, and necrotic tissue (**Figure 3C**). Notably, across the analyzed nasal biopsies, NETs were detected exclusively along the biopsy borders and were absent from the deeper tissue interstitium. This consistent spatial pattern suggests a localized peripheral (i.e. superficial) distribution rather than a diffuse presence throughout the tissue.

### NETs are exclusively detected in perifungal necrotic plaques and do not colocalize with *Aspergillus* conidia and hyphae

**Figure 4:**
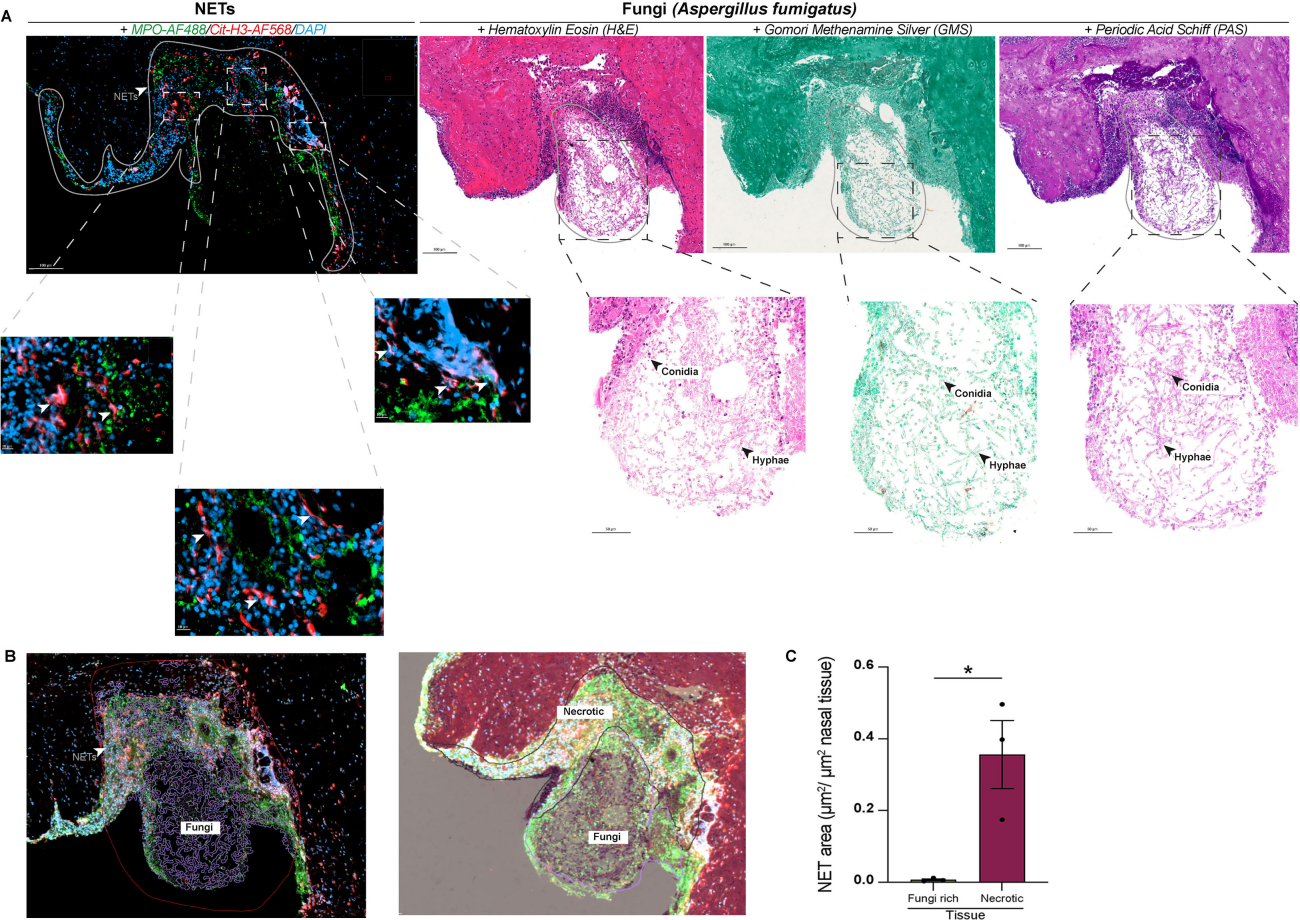
NETs-enriched areas surround fungus rich tissue lesions **(A)** Representative confocal microscopy images of nasal tissue biopsy stained with anti-MPO (green) and anti-citrullinated histone H3 (Cit-H3, red) antibodies, with DAPI (blue) counterstaining nuclei. Serial sections of the nasal biopsy stained for NETs markers, Hematoxylin and Eosin (H&E). **(B, C)** Quantification of NETs formation in ROI, regions of interest of the tissue biopsy, expressed as NETs area per tissue unit. White arrows denote NETs-positive areas identified by immunofluorescence. Scale bars appear below each panel. Data are presented as mean ± s.e.m., with individual values shown. Statistical analysis was performed using a one-way ANOVA with Kruskal-Wallis *post hoc* test. *P < 0.05; **P < 0.01; ***P < 0.001; ns, not significant Tables.

Finally, we investigated the spatial relationship between NETs-enriched areas and fungal elements. Fungal elements morphologically consistent with *Aspergillus* spp. were identified in serial histological sections of nasal tissues from dogs with initial SNA. Sections were stained with Periodic Acid–Schiff (PAS) and Grocott Methenamine Silver (GMS), while Hematoxylin–Eosin (H&E) provided an overview of the inflammatory response (33, 34) (**Figure 4A**). In agreement with Vincent et al. (33), GMS exhibited the highest sensitivity for fungal detection (80–98%), followed by PAS (70–90%), whereas H&E displayed markedly lower sensitivity (50–80%). Lesional areas containing fungal structures were delineated using H&E, GMS and PAS sections, and tissue components including necrotic areas, fungal spores, and hyphae were segmented using QuPath’s machine learning–based pixel classification. To quantify NETs formation, PAS-stained sections were co-registered with immunofluorescence slides using the Warpy extension. NETs were defined by the colocalization of MPO, Cit-H3, and DAPI signals, and a custom analysis pipeline was applied whereby each fluorescence channel was independently thresholded before generating a new channel representing the intersection of all three. This allowed surface quantification of triple-positive voxels, normalized to the total tissue surface measured in each acquisition field. Spatial comparison of NET signals with fungal reference stainings demonstrated that NETs were exclusively detected in perifungal necrotic plaques and were absent from regions containing *Aspergillus* conidia and hyphae (**Figure 4B**). Using serial sections from the same individuals, regions of interest (ROIs) corresponding to fungi-rich tissue and adjacent necrotic tissue were defined based on histological features and transferred onto immunofluorescence images for quantitative assessment. Consistent with microscopic observations, normalized NETs area was significantly higher in necrotic tissue compared with fungi-rich areas (**Figure 4C**).

## Discussion

In dogs, SNA is among the most common causes of chronic nasal discharge, with *Aspergillus fumigatus* representing the primary etiological agent (18). Despite its frequency, the mechanisms governing host defense and the variable clinical course of SNA remain poorly understood, limiting the development of targeted therapeutic approaches.

This study included dogs referred to the university veterinary clinic, a recruitment method that imposed several constraints. Control dogs are rarely evaluated in such settings; thus, we enrolled one experimental beagle dog from the University of Liège teaching kennel and client-owned dogs, all euthanized for unrelated, non-upper respiratory conditions. All control dogs were considered healthy at the level of the upper respiratory tract based on history, the absence of clinical signs and normal physical examination. To preclude potential post-mortem deterioration and its subsequent influence on NETs levels, serum and nasal samples were collected immediately prior to euthanasia. Dogs With SNA were all client-owned dogs referred for chronic nasal disease. Final diagnosis was based on history, clinical signs, head CT if available, typical endoscopic findings, visualization of fungal plaques and identification of *Aspergillus fumigatus* based on fungal culture and/or qPCR. Additionally, dogs diagnosed with SNA typically receive effective treatment at the initial visit, and only a minority return for follow-up. In these cases, clinical improvement and progressive healing of the nasal cavity limited the feasibility of obtaining additional biopsies. No significant statistical differences were detected in Cf DNA or MPO–DNA complexes within the nasal lavage fluid between initial, persistent and resolved cases of SNA. The lack of significance is most likely attributable to the mentioned limitations, particularly the small sample sizes in the resolved and persistent groups, which included only two resolved and four persistent cases. These constraints stem from the inherent difficulty of recruiting and sampling dogs at these specific disease stages. Expanding the cohort in future investigations will be essential to increase statistical power and further evaluate the observed trend. Consequently, the resolved category was excluded from nasal NETs area quantification because it contained only a single available biopsy. This limitation reflects the practical challenges inherent to longitudinal sampling in clinical veterinary contexts, particularly when repeated invasive procedures are ethically and logistically difficult to justify.

We characterized NETs presence in sino-nasal fluids and biopsies from dogs with aspergillosis and compared them with dogs exhibiting persistent or resolved disease and with healthy controls. Three complementary methodological approaches were used. First, cf DNA quantification by Quant-iT PicoGreen allowed rapid assessment but is intrinsically nonspecific, as cf DNA can originate from cellular injury or apoptosis. Second, measurement of MPO–DNA complexes via a previously optimized ELISA (high-throughput, semi-specific) provided an additional surrogate marker for NETs. Although some debate persists regarding the specificity of MPO–DNA (35, 36), given that MPO, as a positively charged secreted protein, can bind negatively charged cf DNA released during non-NET cell death the consistency of results across distinct sample types strengthened the conclusion that detected signals reflected genuine NETs. Third, confocal microscopy enabled direct visualization of filamentous extracellular structures and provided quantitative assessment of NET area using an unbiased computational pipeline based on strict co-localization of MPO (green), citrullinated histone H3 (red), and DAPI-stained DNA (blue). In our view, this approach approximates the gold standard for NET identification in tissues, especially when histone citrullination is present, as MPO^+^/Cit-H3^+^ extracellular filaments strongly support NETosis (8, 29, 35, 37, 38).

Fungal structures were found exclusively at the tissue periphery and did not infiltrate the mucosa, as described in previous studies (39–42). Morphological preservation of *Aspergillus* elements was occasionally compromised by processing steps required for confocal imaging. NETs were consistently observed in biopsies from dogs with SNA, and in four samples we successfully identified preserved *A. fumigatus* hyphae and conidia located in close proximity to NETs. The loosely attached appearance of fungal elements implies they may detach during sample handling, consistent with their superficial localization. Sampling location within the nasal cavity may have influenced fungal detection, as clinicians must avoid hemorrhagic or inaccessible regions. Nonetheless, the integration of biopsy imaging with biological fluid analysis (e.g., NALf) mitigated potential site-related biases.

Clinical data already showed that complete MPO deficiency in humans primarily results in recurrent fungal infections (43). This is supported by studies in MPO-deficient mice, which suggest a crucial role for NETs against pathogens too large for intracellular killing, such as fungal hyphae (25). Furthermore, the importance of NETs in resolving systemic fungal infection is evidenced by the restoration of NETosis in a Chronic Granulomatous Disease patient following gene therapy (44). Consistent with this models, human and murine studies have demonstrated that NETs can restrict the spread of *A. fumigatus* (24, 26). McCormick et al. showed that conidia and germ tubes trigger NET formation, even without fungal viability. Neutrophils phagocytose conidia and suppress germination through mechanisms independent of NETosis, whereas germ tubes resist killing but exhibit reduced polar growth in a NET- and DNase-I-sensitive manner. Calprotectin, a zinc-binding protein associated with NETs, contributes to this inhibitory effect, and Zn²^+^ supplementation reverses growth restriction. Our observations in canine sinonasal aspergillosis parallel these findings: NETs were detected exclusively at the nasal biopsy margins and were associated with fungal-rich regions, strongly supporting the hypothesis that NETs primarily limit fungal dissemination rather than exert direct fungicidal activity.

To date, only a limited number of studies have documented NETs in dogs (21, 45). NETs were first described in canine tissue in a 2025 report implicating them in thrombosis associated with bacterial vasculitis, confirmed via immunofluorescent co-localization of MPO, Cit-H3, and DNA, and supported by Cit-H3 immunohistochemistry (46). Additional studies have identified NETs in contexts such as sepsis and immune thrombosis (47, 48). In septic dogs, cf DNA and nucleosomes were elevated relative to controls, particularly in cases of bacteremia, suggesting intravascular NETosis and potential diagnostic value for point-of-care cf DNA (47). NET release has also been demonstrated *in vitro* following stimulation of canine neutrophils with *Escherichia coli* (49) and in cytological samples from septic foci (48). However, no previous study has documented NETs within the canine respiratory system or investigated their role in fungal infections. Our work is, to our knowledge, the first to apply two highly specific NET-detection strategies in this context: (1) ELISA quantification of MPO–DNA complexes in NALf and serum, and (2) histological NET area assessment using MPO/Cit-H3/DNA co-localization on confocal microscopy images. These complementary methods provide robust tools for evaluating NET formation in both fluids and tissues.

Because *A. fumigatus* is considered non-invasive in the human respiratory tract (26), we hypothesized that NET release in canine SNA would be locally restricted rather than systemic. Consistent with this, serum analyses revealed no significant differences in NET markers among SNA groups or compared with controls. These findings support the conclusion that NET formation in SNA is confined to the nasal mucosa and does not trigger widespread systemic NETosis.

The precise contribution of NETs to the progression of sinonasal aspergillosis is still under investigation. DNA extracellular traps are formed in experimental pulmonary aspergillosis and require neutrophils for *in vivo* induction (24). Neutrophils do not directly kill conidia but inhibit germination both *in vitro* (22, 26) and *in vivo* (50), partly through NET-associated sequestration and metal chelation. Yet Gazendam et al. (22) reported no essential role for NETs in fungicidal activity, attributing inhibition instead to lactoferrin and calprotectin release, while hyphal killing depended on NOX2-mediated oxidative bursts. Despite these discrepancies, our results add a new perspective by showing that NETs are present early in canine SNA. Morphological assessment suggests that their primary function is not to directly kill the fungus but rather encapsulate and physically contain fungal elements, thereby restricting dissemination within the nasal cavity even in the absence of robust fungicidal activity. This localized containment mechanism likely explains the consistent presence of NETs in initial disease stages. Although the NET induction pathway in SNA remains unknow, it is consistent with previous mechanistic studies in which NET release in response to *A. fumigatus* is known to depend on CD11b/CD18 (Mac-1) but not on activation of TLR2, TLR4, or Dectin-1 (51). Understanding whether similar receptor-dependent pathways operate in canine neutrophils warrants further investigation. Extensive and irreversible turbinate destruction is thought to predispose affected dogs to chronic lymphoplasmacytic rhinosinusitis. The persistence of NETs within the nasal mucosa and/or secretions post-cure could suggest a role for NETs in sustaining secondary chronic inflammation. Indeed, prolonged or excessive NETosis may contribute to tissue damage and delay the repair of non-infected epithelium (4, 52).

Excessive neutrophil infiltration and NET release are widely implicated in tissue injury across multiple conditions, including acute lung injury, myocardial infarction, stroke, and organ failure (53–55). These detrimental effects are typically attributed to dysregulated NETosis, ROS generation, and cytotoxic granule release. In contrast, the present study suggests a beneficial role for NETs in dogs with SNA. Our observations support that NETs limit the spread of fungal hyphae and conidia at the site of infection, thereby acting as a localized structural defense mechanism rather than a driver of collateral tissue damage.

NETs were detectable only in samples from dogs with initial SNA, not in controls. These findings support that NETs participate to the inflammatory process associated with SNA and may regulate disease progression. Whether NETs primarily contain fungal spread or play a pathogenic role remains unclear, but their selective presence in SNA highlights their potential as future therapeutic targets.

These findings reveal that, during initial SNA, neutrophils exhibit a marked propensity to release NETs. However, as NETs accumulate primarily in necrotic regions rather than within fungal-rich areas, their role may be associated with fungal sequestration rather than direct host tissue damage. As these represent initial observations in dogs, further studies are required to confirm the exact functional role of NETs in the pathogenesis of this disease.

## Data Availability

The original contributions presented in the study are included in the article/supplementary material. Further inquiries can be directed to the corresponding authors.
